# Exclusive breastfeeding among HIV exposed infants from birth to 14 weeks of life in Lira, Northern Uganda: a prospective cohort study

**DOI:** 10.1080/16549716.2020.1833510

**Published:** 2020-10-30

**Authors:** Agnes Napyo, James K. Tumwine, David Mukunya, Paul Waako, Thorkild Tylleskär, Grace Ndeezi

**Affiliations:** a Department of Public Health, Faculty of Health Sciences, Busitema University, Tororo, Uganda; b Centre for International Health, University of Bergen, Bergen, Norway; c Department of Pediatrics and Child Health, Makerere University, Kampala, Uganda; d Department of Pharmacology, Faculty of Health Sciences, Busitema University, Tororo, Uganda

**Keywords:** Breastfeeding, exclusive breastfeeding, HIV exposed infants, infant feeding, mixed feeding, Women living with HIV

## Abstract

**Background:**

Breastfeeding is important for growth, development and survival of HIV exposed infants. Exclusive breastfeeding reduces the risk of morbidity, mortality and increases HIV free survival of infants. Evidence on risk factors for inappropriate breastfeeding in Northern Uganda is limited.

**Objective:**

This study determined the risk factors for non-exclusivity of breastfeeding in the first 14 weeks of life.

**Methods:**

This prospective cohort study was conducted among 466 mother-infant pairs between August 2018 and February 2020 in Lira district, Northern Uganda. HIV infected pregnant women were enrolled and followed up at delivery, 6- and 14- weeks postpartum. We used a structured questionnaire to obtain data on socio-demographic, reproductive-related, HIV-related characteristics and exclusive breastfeeding. Data were analysed using Stata version 14.0 (StataCorp, College Station, Texas, USA.). We estimated adjusted risk ratios using modified Poisson regression models.

**Results:**

The proportion of HIV exposed infants that were exclusively breastfed reduced with increasing age. Risk factors for non-exclusive breastfeeding included infants being born to HIV infected women who: were in the highest socioeconomic strata (adjusted risk ratio = 1.5, 95%CI: 1.01– 2.1), whose delivery was supervised by a non-health worker (adjusted risk ratio = 1.6, 95%CI: 1.01– 2.7) and who had not adhered to their ART during pregnancy (adjusted risk ratio = 1.3, 95%CI: 1.01– 1.7).

**Conclusions:**

HIV infected women: with highest socioeconomic status, whose delivery was not supervised by a health worker and who did not adhere to ART were less likely to practice exclusive breastfeeding. We recommend ART adherence and infant feeding counselling to be emphasised among HIV infected women who are at risk of having a home delivery, those with poor ART adherence and those of higher socioeconomic status. We also recommend integration of these services into other settings like homes, community and work places instead of limiting them to hospital settings.

**Abbreviations:**

HIV: Human Immunodeficiency Virus; ART: Antiretroviral therapy; HEI: HIV exposed infant; PMTCT: Prevention of mother-to-child transmission of HIV; MTCT: Mother-to-child transmission of HIV; AFASS: Acceptable, Feasible, Affordable, Sustainable and Safe; LRRH: Lira regional referral hospital; CI: confidence interval; ARR: Adjusted risk ratio; SD: Standard deviation; PCA: Principal component analysis

## Background

Breastfeeding is important for growth, development and survival in children [1]. In HIV exposed infants (HEIs), exclusive breastfeeding is very important because they are more prone to diarrhoea, pneumonia, malnutrition and even death [2] compared to unexposed infants. Various studies have demonstrated the positive impact of breastfeeding on child survival. A number of studies have shown the benefits of breastfeeding which include: lowered risk of incidence and death from infections like diarrhoea and pneumonia [3,4] as well as reduced risk of hospitalisation [5]. Mixed feeding, when compared with exclusive breastfeeding, increases the risk of morbidity and HIV transmission by four fold [6,7] in addition to reducing HIV free survival among HEI [3]. Human breast milk contains various immunoglobulins, proteins, hormones, growth factors, lipids, carbohydrates and microbiota that play a very important role in the immunomodulation, immune-regulation as well as defence against pathogenic bacteria and viruses for the infant/neonate [8]

Guidelines from the World Health Organisation [1,9] and the Ugandan Ministry of Health [10] recommend that an HIV infected woman should breast feed her baby exclusively for the first 6 months and continue breastfeeding till the baby is 12–24 months of age while introducing appropriate complimentary foods. In cases where exclusive breastfeeding is not possible, exclusive replacement feeding is recommended provided it follows the AFASS criteria meaning it should be Acceptable, Feasible, Affordable, Sustainable and Safe. Women living with HIV who are lactating should take antiretroviral therapy (ART) regardless of their CD4 count and should be adherent to it [1]. ART helps to prevent HIV transmission during the phase of exclusive breastfeeding in the first 6 months of life and also during mixed feeding thereafter [11].

Barriers to breastfeeding include maternal factors like breast problems, home delivery [12], lack of safe water, cultural beliefs [13] and lack of counselling or support during continuation of infant feeding [6] as well as infant factors such as mouth ulcers [11]. Interventions such as counselling tend to improve exclusive breastfeeding rates [3], however, their implementation has to be broad based and should cover a wide range of settings such as homes, health facilities, communities and work places so as to yield a higher impact on breastfeeding.

Several studies have shown low rates of exclusive breastfeeding among HEI [6,11,12,14,15,16]. Risk factors for these low rates are not well understood and vary with in different contexts. The settings for these studies have been heterogeneous and tend to vary from country to country. Very few studies have been done in Uganda, particularly Northern Uganda. There is limited information on risk factors for inappropriate breastfeeding by HIV infected women in Lira, Northern Uganda. Therefore, our study aimed to determine the risk factors for non-exclusive breastfeeding in the first 14 weeks of life among HEIs.

## Methods

### Study design and setting

A prospective cohort study was done in Lira, Northern Uganda between August 2018 and February 2020 at the Prevention of Mother-to-Child Transmission (PMTCT) of HIV clinic in the Lira Regional Referral Hospital (LRRH). Lira is home to over 400,000 people who are predominantly Langi. It has a diversified economy characterised mainly by farming, brick making, boda boda (motorcycle) public transportation and pottery [17]. Northern Uganda, particularly Lira, has a very high antenatal HIV prevalence of 13.5% [18] which directly translates into a higher risk of mother-to-child transmission (MTCT) of HIV. The PMTCT clinic is an initiative of the Ugandan Ministry of Health where free HIV care and treatment is offered to HIV-infected pregnant and lactating women. Within this clinic, antenatal care is offered for HIV infected pregnant women every day of the week with the exception of weekends. On a daily basis, health education is given in group sessions only early in the morning to mothers who have come for antenatal care. Health education topics include infant feeding in the context of HIV, adherence to ART, viral load testing, maternal nutrition and malaria prevention.

### Participants and procedures

This study involved HIV infected women and their infants. At baseline, HIV infected pregnant women who were receiving antenatal care at LRRH with a gestational age of 20 weeks or more were consecutively enrolled onto the study and interviewed on socio-demographic and HIV-related information. A viral load test was done during any stage of pregnancy. The date of delivery was estimated using the palpation method, gestational wheel and first day of the last normal menstrual period. These women were then followed up with a telephone interview around the time of delivery. This follow-up visit was estimated at 7 days after the expected date of delivery. If the woman had not delivered yet, another telephone appointment was scheduled. At this point, women were interviewed on circumstances surrounding labour and delivery such as time of onset of labour, type of delivery, place of delivery, person who supervised the delivery, if the baby received any prelacteal feeds as well as maternal ART adherence. At 6 weeks postpartum, mothers were followed up with a face-to-face interview and asked about the infant’s adherence to nevirapine prophylaxis and exclusivity of breastfeeding. When the infant was 14 weeks of age, women were also asked about exclusivity of breastfeeding through a face-to-face interview. We used a 7-day recall for and obtained information about exclusivity of breastfeeding at the different follow up points from when the infant was born to 14 weeks of age [19]. All study follow-up visits with the exception of delivery were done at the PMTCT clinic. The study visits were conveniently planned to coincide with the mothers’ ART refills and the infants’ immunisation schedule. We scheduled the study visits this way so that the mothers did not have to make extra visits to the clinic just for purposes of the study and in so doing we saved on extra transportation costs for the participants.

### Sample size estimation

We calculated a sample size for detecting a difference between two independent proportions using STATA version 14.0 (StataCorp; College Station, TX, USA). We assumed 80% power, 95% confidence interval (CI) and a 5% precision. We also assumed that 70% of women [20] received EBF support and counselling at delivery and that 42.5% of women were not advised or counselled on exclusive breastfeeding during pregnancy [21]. We made these assumptions in order obtain the minimum sample size required to detect a difference between exclusive breastfeeding and non-exclusive breastfeeding. The total sample size for this study was 418 HEI. After accounting for 10% non-response the final sample size was 464. We however, included 466 HEI.

### Measurement of variables

Prelacteal feeding was defined as the baby feeding on any liquid other than breast milk immediately after birth with the exception of medicines like nevirapine syrup. The mother was asked, ‘After delivery, did you give the baby anything before giving him/her breast milk?’ This was a ‘yes’ or ‘no’ response. We, however, did not ask which liquids had been given to the baby as pre-lacteal feeds. For both the 6-week and 14-week visits, the mother was asked, ‘In the past week, have you given the baby any liquid or solid food other than the breast milk?’ This was also a ‘yes’ or ‘no’ response. If the mother’s response was ‘yes’, then she was asked, ‘What food or liquid did you feed to the baby’. The baby was considered to be non-exclusively breastfed if the mother had reported giving prelacteal feeds at birth or if the mother had reported giving liquids or solid food other than breast milk at the 6-week and 14-week visits with the exception of medicines such as nevirapine or supplements like multivitamins.

We also measured maternal ART adherence during the follow-up at the time of delivery, we asked the mother, ‘In the past week, did you miss taking any dose of your medication?’ This was a ‘yes’ or ‘no’ response. If the mother answered ‘yes’ she was considered ‘non-adherent’. We also asked who had assisted her during delivery and if she responded that it was a nurse, doctor, student nurse, clinical officer, midwife, all these responses were categorised into one group and labelled ‘birth supervised by health worker’. If the response was ‘mother-in-law, traditional birth attendant, or good samaritan’ all these responses were categorised together and labelled ‘birth supervised by non-health worker’

We created a composite index of wealth (socio-economic status) using principle component analysis (PCA). We used PCA on house ownership, availability of electricity in the house, source of drinking water and fuel used for cooking [22]. Scores were obtained and categorized into three groups which we refer to as strata ranging from the poorest to the least poor.

### Data analysis and management

Data were collected using pretested, structured questionnaires, doubly entered into Epi data (www.epidata.dk, version 4.4.3.1) and exported for analysis to Stata version 14.0 (StataCorp, College Station, Texas, USA.). Only mother-infant pairs with data at the three time points of follow-up were included in the analysis. Continuous data, if normally distributed, was summarised into means and standard deviations and if skewed, was summarised into medians with their corresponding interquartile ranges. Categorical variables were summarised into frequencies and percentages. The incidence of non-exclusivity of breastfeeding was estimated and its confidence limits calculated using the exact method. Bivariable and multivariable analysis was done using the modified Poisson regression model [23]. All variables that had a p value < 0.25 at bivariable level and those with biological plausibility were entered into the multivariable model. Variables that were independently associated with non-exclusivity of breastfeeding were determined using the confidence limits.

## Results

**Figure 1.**
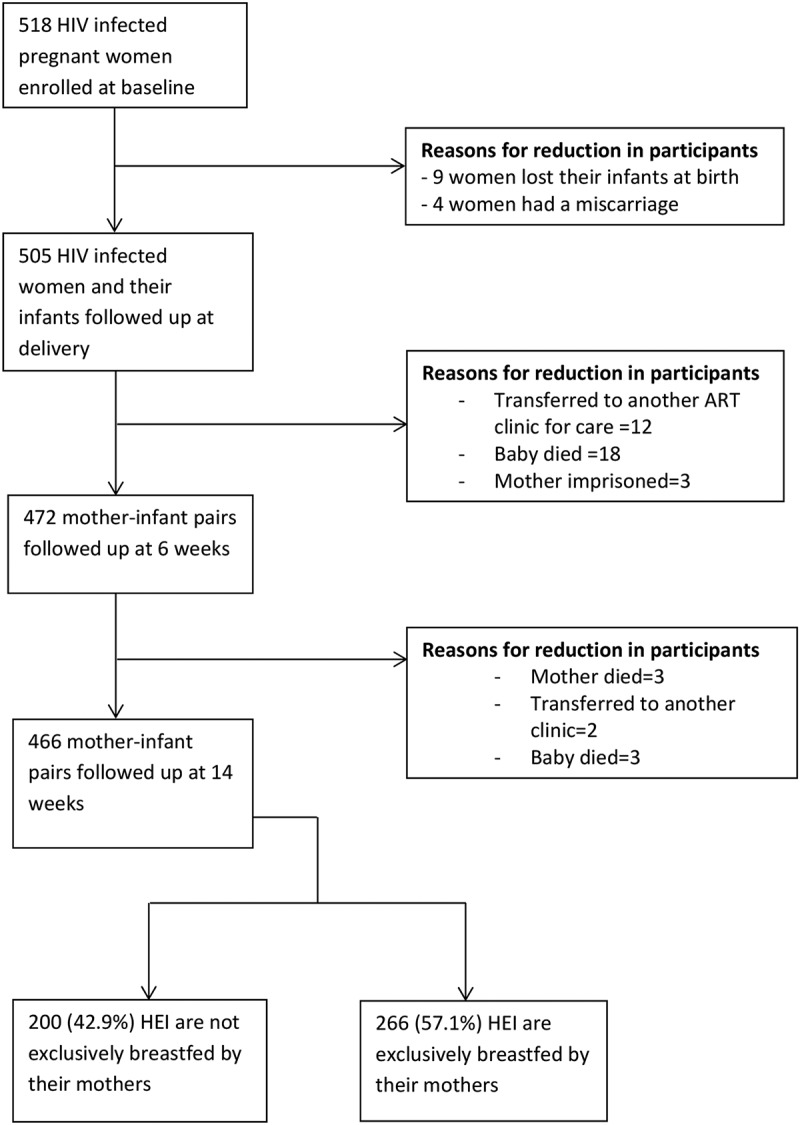
Study flow chart

A total of 518 HIV infected women were enrolled on to the study and followed up till delivery at which point 505 women had given birth to their infants. These women then were followed up till 6 weeks postpartum at which point 472 women and their infants had the required data on exclusivity of breastfeeding. Complete information was obtained for 466 mothers by 14 weeks and these were included in our analysis. The reasons for loss to follow up of participants at subsequent visits are explained in the study flow chart (Figure 1)

### Characteristics of HIV infected pregnant women at baseline

Almost fifty per cent of the women were aged 30 years or more with a mean age of 29.5 years (Standard deviation (SD) 5.4). Most women were married, unemployed and had attained at least 6 years of education (Table 1). More than half had been pregnant four times and were 20– 27 weeks of gestation at enrolment. Most of the women had disclosed their HIV status. A considerable proportion of them were taking an efavirenz-based regimen and more than half had a viral load less than 50 copies/ml.

**Table 1.** Antenatal baseline characteristics for HIV infected women in the study in relation to breastfeeding at 14 weeks postpartum

	Total N = 466 n (%)	Exclusive breastfeeding at 14 weeks postpartum N = 266 n (%)	Not exclusive breastfeeding at 14 weeks postpartum N = 200 n (%)	P-value
**DURING PREGNANCY**				
**Age**				0.5
≤ 20 years	27 (5.8)	13 (4.9)	14 (7)	
21– 29 years	202 (43.3)	120 (45.1)	82 (41)	
≥ 30 years	237 (50.9)	133 (50)	104 (52)	
**Education status**				0.9
≤ 6 years	229 (49.1)	131 (49.3)	98 (49)	
7– 13 years	166 (35.6)	96 (36.1)	70 (35)	
≥ 14 years	71 (15.3)	39 (14.6)	32 (16)	
**Marital status**				0.9
Married	435 (93.4)	248 (93.2)	187 (93.5)	
Single	31 (6.6)	18 (6.8)	13 (6.5)	
**Employment status**				0.1
Employed	189 (40.6)	100 (37.6)	89 (44.5)	
Unemployed	277 (59.4)	166 (62.4)	111 (55.5)	
**Religion**				0.04
Christian	448 (96.1)	260 (97.7)	188 (94)	
Moslem	18 (3.9)	6 (2.3)	12 (6)	
**Ethnic belonging**				0.8
Lango	424 (90.9)	243 (91.4)	181 (90.5)	
Other	42 (9.1)	23 (8.6)	19 (9.5)	
**Parity**				0.8
1 to 4	329 (70.6)	189 (71.1)	140 (70)	
5 to 9	137 (29.4)	77 (28.9)	60 (30)	
**Gestational age**				0.3
20– 27 weeks	243 (52.2)	133 (50)	110 (55)	
28– 35 weeks	158 (33.9)	98 (36.8)	60 (30)	
≥ 36 weeks	65 (13.9)	35 (13.2)	30 (15)	
**HIV disclosure**				0.8
Disclosed	451 (96.8)	257 (96.6)	194 (97)	
Not disclosed	15 (3.2)	9 (3.4)	6 (3)	
**Socioeconomic strata**				0.06
Lowest	164 (35.2)	105 (39.5)	59 (29.5)	
Middle	147 (31.5)	82 (30.8)	65 (32.5)	
Highest	155 (33.3)	79 (29.7)	76 (38)	
**Antiretroviral regimen**				0.7
Efavirenz-based	420 (90.1)	237 (89.1)	183 (21.5)	
Nevirapine-based	38 (8.2)	24 (9.0)	14 (7)	
Protease inhibitor-based	8 (1.7)	5 (1.9)	3 (1.5)	
**Antiretroviral treatment duration**				0.08
≤ 6 months	82 (17.6)	46 (17.3)	36 (18)	
7– 30 months	101 (21.7)	50 (18.8)	51 (25.5)	
31– 119 months	251 (53.8)	146 (54.9)	105 (52.5)	
≥ 120 months	32 (6.9)	24 (9)	8 (40)	
**Viral load count**				0.7
<50 copies/ml	264 (56.8)	150 (56.6)	114 (57)	
50– 400 copies/ml	76 (16.3)	43 (16.2)	33 (16.5)	
401– 499 copies/ml	12 (2.6)	8 (3)	4 (2.0)	
>1000 copies/ml	27 (5.8)	18 (6.8)	9 (4.5)	
missing viral load	86 (18.5)	46 (17.4)	40 (20.0)	

### Characteristics at birth and 6 weeks postpartum

Most of the mothers had their labour start during the day time and had a spontaneous vaginal delivery (Table 2). Three quarters of them delivered in a hospital setting with most deliveries being supervised by a health worker. Almost a third of these women did not adhere to their antiretroviral treatment in the week preceding delivery. Thirty per cent of 6-week old infants missed receiving one or more doses of the nevirapine prophylaxis from their mother or caregiver.

**Table 2.** Characteristics for HIV infected pregnant women and HIV exposed infants at the time of delivery and 6 weeks postpartum

	Total N = 466 n (%)	Exclusive breastfeeding at 14 weeks postpartum N = 266 n (%)	Mix feeding at 14 weeks postpartum N = 200 n (%)	P-value
**AT DELIVERY**				
**Time of onset of labour**				0.5
Day time	243 (52.2)	135 (50.8)	108 (54)	
Night time	223 (47.8)	131 (49.2)	92 (46)	
**Type of delivery**				0.06
Spontaneous vaginal	409 (87.8)	240 (90.2)	169 (84.5)	
Caesarean section	57 (12.2)	26 (9.8)	31 (15.5)	
**Place of delivery**				0.3
Hospital setting	435 (93.4)	251 (94.4)	184 (92)	
Non hospital setting	31 (6.6)	15 (5.6)	16 (8)	
**Person who supervised delivery**				0.05
Health worker	436 (93.6)	254 (95.5)	182 (91)	
Non health worker	30 (6.4)	12 (4.5)	18 (9)	
**Infant given prelacteal feeds**				0.000
Yes	59 (12.7)	0 (0)	59 (29.5)	
No	407 (87.3)	266 (100)	141 (70.5)	
**Maternal adherence to** **antiretroviral treatment**				0.06
Adhered	325 (69.9)	195 (73.3)	130 (65.3)	
Did not adhere	140 (30.1)	71 (26.7)	69 (34.7)	
**AT 6 WEEKS POSTPARTUM**				
**Infant adherence to nevirapine prophylaxis**				0.03
Adhered	317 (68.1)	192 (72.2)	125 (62.5)	
Did not adhere	149 (30.1)	74 (27.8)	75 (37.5)	
**Infant exclusive breastfeeds at 6 weeks**				0.000
Exclusively breastfed	361 (77.5)	266 (100)	95 (47.5)	
Mixed feeding	105 (22.5)	0 (0)	105 (52.5)	

### Infant feeding practices at delivery, 6 and 14 weeks postpartum

**Figure 2.**
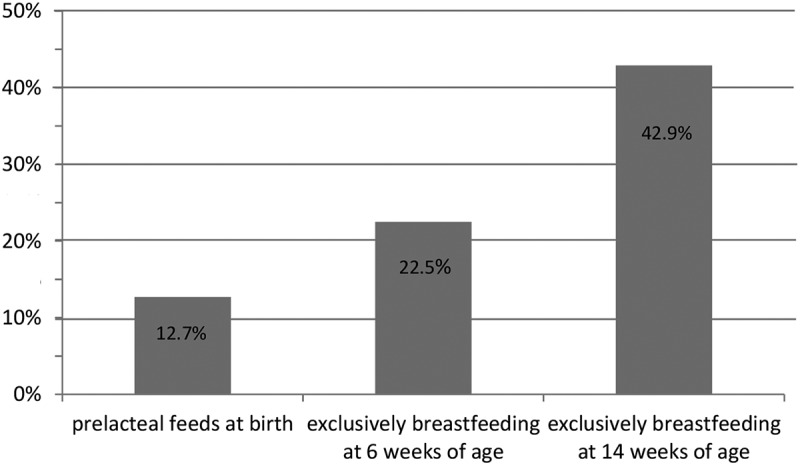
Infant feeding practices among HIV exposed infants by HIV infected lactating women

The proportion of infants that were exclusively breastfed reduced with increasing age of the infant (Figure 2). Incidence of pre-lacteal feeding at birth was 12.7% (95%CI: 9.8–16%) (Table 2). The incidence of non-exclusivity of breastfeeding at 6 weeks and 14 weeks postpartum were 22.5% (95%CI: 18.8–26.6%) and 42.9% (95%CI: 38.3–47.5%) respectively (Table 3). By the time the infants were 14 weeks of age, almost half of them were not exclusively breastfeeding (Table 3).

**Table 3.** Feeds given to HIV exposed infants at 6 weeks and 14 weeks postpartum

	Feeds given to infants at 6 weeks postpartum (N = 466)		Feeds given to infants at 14 weeks postpartum (N = 466)	
Type of infant’s feed	**n**	**%**	**n**	**%**
Only breast milk	361	77.5	266	57.1
Honey	55	11.8	44	9.4
Water	23	4.9	36	7.7
Cow’s milk	13	2.8	70	15
Soup	6	1.3	17	3.7
Porridge	5	1.1	21	4.5
Infant formula	3	0.6		
Juice			11	2.4
Solid food			1	0.2

### Risk factors for non-exclusivity of breast feeding at 14 weeks of age

Women who were in the highest socioeconomic strata were 50% more likely to give their infants liquids other than breast milk when compared to those in the lowest socioeconomic strata (Adjusted Risk ratio (ARR) = 1.5, 95%CI: 1.01– 2.1). Women whose delivery was supervised by a non-health worker were 60% more likely to practice mixed feeding when compared to those whose delivery had been supervised by a health worker (ARR = 1.6, 95%CI: 1.01– 2.7). Women who had not adhered to their ART during pregnancy were also likely to practice mixed feeding for their infants when compared to their adherent counterparts (ARR = 1.3, 95%CI: 1.01– 1.7) (Table 4).

**Table 4.** Risk factors for non-exclusivity of breastfeeding among HIV exposed infants at 14 weeks postpartum

	Unadjusted RR (95% CI)	Adjusted RR (95% CI)
**Age**		
≤ 20 years	1.2 (0.7 – 2.1)	1.1 (0.6 – 2)
21 – 29 years	0.9 (0.7 – 1.2)	0.8 (0.6 – 1.2)
≥ 30 years	1	1
**Education status**		
≤ 6 years	1	1
7 – 13 years	1.0 (0.7 – 1.3)	0.9 (0.7 – 1.3)
≥ 14 years	1.1 (0.7 – 1.6)	1.01 (0.7 – 1.5)
**Parity**		
0 to 4	1	1
5 to 9	1.01 (0.8 – 1.4)	1.0 (0.7 – 1.4)
**Socioeconomic status**		
Lowest	1	1
Middle	1.2 (0.9 – 1.8)	1.3 (0.9 – 1.8)
Top	1.4 (0.9 – 1.9)	**1.5 (1.01 – 2.1)**
**Antiretroviral treatment duration**		
≤ 6 months	1	1
7 – 30 months	1.2 (0.8 – 1.8)	1.27 (0.8 – 2)
31 – 119 months	0.9 (0.7 – 1.4)	1.0 (0.7 – 1.6)
≥ 120 months	0.6 (0.3 – 1.2)	0.6 (0.3 – 1.3)
**Person who supervised delivery**		
Health worker	1	1
Non health worker	1.4 (0.9 – 2.3)	**1.6 (1.01 – 2.7)**
**Maternal adherence to antiretroviral treatment**		
Adhered	1	1
Did not adhere	1.2 (0.9 – 1.7)	**1.3 (1.01 – 1.7)**

## Discussion

The proportion of infants that were exclusively breastfed reduced with increasing age of the infant and by 14 weeks of age, almost half of the infants were not exclusively breastfeeding. We found a low incidence of EBF among 14 week-old HEIs probably because their mothers perceive that their breast milk is so insufficient that it will not satisfy the baby and so resort to other foods as feeding options for the infant such as cow’s milk, water and porridge [21]. Cultural beliefs surrounding breastfeeding also influence infant feeding practices, for example, believing that giving the baby honey will protect them against false teeth and colic pain [24]. Giving the baby prelacteal feeds also contributed to the incidence of non-EBF. Studies from South Africa [6] and Nigeria [14] report a similar trend in exclusive breastfeeding among HIV exposed infants as they grow older. Some systematic reviews [12,16] and observational studies [11,15] have reported rates similar to that in our study. However one study from Tanzania [25] reported a higher prevalence of exclusive breastfeeding than our study. These disparities could be explained by the fact that all these studies included infants of varying ages and were done in different socio-cultural contexts.

It was common for mothers to give their infants prelacteal feeds after delivery. Women are most likely to give their babies prelacteal feeds because of sore breasts, perceived insufficient milk flow immediately after delivery, social and cultural issues like discarding of colostrum [21]. Several studies of HEI infants [11] demonstrate that mothers give infants these feeds due to insufficient breast milk shortly after delivery, because of breast problems or maternal death. One study from Northern Uganda, a context similar to our study, showed that lactating women discard colostrum shortly after delivery because they culturally perceive it to be dirty and harmful to the baby [21]. This could possibly explain why infants in our cohort were given prelacteal feeds. An infant missing out on colostrum misses out on the essential benefits like building up of their immune system and lining of the infant’s gut to keep pathogens at bay. This is a potential risk for mother-to-child transmission of HIV and development of opportunistic infections.

In our cohort, women of the highest socioeconomic status were more likely not to exclusively breastfeed their infants when compared to those in the lowest socioeconomic strata. One study [26] demonstrated an association between socioeconomic status and exclusive breast feeding. Most women in the top most socioeconomic strata in our cohort were actually employed and probably had to return to work shortly after delivery because of work-related demands and pressures. Furthermore, because of work-related demands these women are more likely not to receive adequate antenatal care and infant feeding counselling and this could explain the finding in our study. In our study setting, infant feeding counselling has also not been integrated with work-place environments. Therefore, women with busy work schedules are less likely to receive infant feeding counselling.

Women whose delivery was supervised by a non-health worker were less likely to exclusively breastfeed and were likely to have had a home delivery. Having a home delivery deprives the mother of interfacing with the health worker and healthcare there by losing out on the benefits of counselling and support for exclusive breastfeeding. Some systematic reviews [3,12,16] and observational studies [11,15,21] showed that women who attended antenatal care clinics, those that delivered in a hospital and those that had infant adherence counselling were more likely to practice exclusive breastfeeding. In a hospital setting, there is on-going infant feeding training for the healthcare worker and infant feeding counselling for the mother. Another study from Northern Uganda [24] found that health workers were key decision makers when it came to breastfeeding. These findings from various studies clearly explain why a mother whose delivery is not supervised by a health worker is most likely not to exclusively breastfeed her infant. In light of this, it is important that infant feeding counselling is introduced in a combination of settings and not only at health facilities: such as at the facility, work place, community and home settings.

Our study demonstrated that women who had not adhered to ART during pregnancy were also likely not to exclusively breastfeed their infants. Being non-adherent to ART is an aftermath of not interfacing routinely with the healthcare system [27]. Therefore, these women will not achieve the benefits of this routine interaction with the healthcare system like continued counselling on infant feeding. Women who are non-adherent to ART will hence most likely not be adherent to infant feeding guidelines and will not exclusively breastfeed their infants. Non-adherence to ART will lead to higher viral loads and advanced HIV disease which poses a high risk for transmission of HIV from a mother to her baby during breastfeeding. Few studies have examined antiretroviral adherence during pregnancy and its association with infant feeding practices. However, pregnancy in itself has been associated with low ART adherence [28]. Drug-related factors such as side effects and pill burden as well as physiological changes during pregnancy are barriers to ART adherence [29]. More qualitative studies should be done to shed more light on the association between ART adherence and breastfeeding.

### Strengths and limitations

This study had some strength. The fact that this is a prospective cohort study has helped to establish causality between various covariates and non-exclusivity of breastfeeding. Most studies that have been conducted on this subject matter have been cross-sectional in nature and only show associations. Showing causality for inappropriate infant feeding paves the way to the designing of interventions to promote exclusive breastfeeding. We also measured exclusive breastfeeding continuously from birth and also relied on a 7-day recall which is likely to avoid exaggeration of the incidence of exclusive breastfeeding in our cohort. To minimize loss to follow-up, we documented the telephone contacts and residential mapping of each participant. Instances where we could not reach the participant on phone, we made a home visit and this resulted into a high completion rate of 94%.

Our study had some limitations. This study was done among HIV infected women attending a public health facility therefore our findings may not be generalizable to women attending clinics that are private-for-profit and private-not-for profit. We measured exclusive breastfeeding at 6 and 14 weeks using a 7-day recall of the mother or care giver. This can potentially be a source for recall bias.

## Conclusion

The proportion of exclusively breastfed HEI reduced with increase in the infant’s age. HIV infected women with highest socioeconomic status, those whose delivery was not supervised by a health worker and those that who did not adhere to antiretroviral treatment were likely not to exclusively breastfeed their infants. We recommend ART adherence and infant feeding counselling to be emphasised and integrated in a diverse settings such as homes, work places, communities and health facilities.

## Data Availability

The datasets used and/or analysed during the current study are available from the corresponding author on reasonable request.

## Paper context

Exclusive breastfeeding reduces the risk of morbidity, mortality and increases HIV-free survival of infants. Evidence on risk factors for inappropriate breastfeeding in Northern Uganda is limited. We therefore aimed to study risk factors for non-exclusivity of breastfeeding among HIV-exposed infants. Findings from this study have identified high-risk women for inappropriate breastfeeding. These women can now be targeted for counselling during the implementation of infant feeding policies in the context of HIV.
